# The association of insufficient gestational weight gain in women with gestational diabetes mellitus with adverse infant outcomes: A case-control study

**DOI:** 10.3389/fpubh.2023.1054626

**Published:** 2023-02-23

**Authors:** Dabin Huang, Mulin Liang, Bin Xu, Shan Chen, Yan Xiao, Hui Liu, Dan Yin, Jun Yang, Ling Wang, PianPian Pan, Yihui Yang, Wei Zhou, Juncao Chen

**Affiliations:** ^1^Department of Neonatology, Guangzhou Women and Children's Medical Centre, Guangzhou Medical University, Guangzhou, China; ^2^Department of Neonatology, The Fifth Affiliated Hospital of Southern Medical University, Guangzhou, China; ^3^Medical Department, The First People's Hospital of Chenzhou, Chenzhou, China; ^4^Rehabilitation Medicine Center, The First Dongguan Affiliated Hospital, Guangdong Medical University, Dongguan, China; ^5^Advanced Institute of Natural Sciences, Beijing Normal University at Zhuhai, Zhuhai, China

**Keywords:** dietary intervention, gestational diabetes mellitus, gestational weight gain, neonatal complications, risk factors, small-for-gestational age

## Abstract

**Background:**

To investigate the association between insufficient maternal gestational weight gain (GWG) during dietary treatment, and neonatal complications of small-for-gestational-age (SGA) infants born to mothers with Gestational diabetes mellitus (GDM).

**Methods:**

A retrospective case-control study was conducted, involving 1,651 infants born to mothers with GDM. The prevalence of a perinatal outcome and maternal GWG were compared among SGA, adequate- (AGA), and large-for-gestational-age (LGA); association with birth weight and GWG was identified using Pearson's correlation analysis; binary logistic regression was performed to determine the odds ratio (OR) associated with SGA.

**Results:**

In total, 343 SGA, 1025 AGA, and 283 LGA infants met inclusion criteria. The frequency of SGA infants who were siblings (41.7 vs. 4.3 vs. 1.9%) and composite of complications (19.2 vs. 12.0 vs. 11.7%) were higher in SGA infants than in those in AGA or LGA infants group (both *P* < 0.01). GWG and pre-partum BMI were lower among the SGA mothers with GDM group (11.7 ± 4.5 kg, 25.2 ± 3.1 kg/m^2^) than AGA (12.3 ± 4.6 kg, 26.3 ± 3.4 kg/m^2^) or LGA (14.0 ± 5.1 kg, 28.7 ± 3.9 kg/m^2^) mothers with GDM group. Binary logistic regression showed that siblings who were SGA (AOR 18.06, 95% CI [10.83–30.13]) and preeclampsia (AOR 3.12, 95% CI [1.34–7.30]) were associated with SGA, but not GWG below guidelines (*P* > 0.05). The risk of SGA (25.7 vs. 19.1 vs. 14.2%) and FGR (15.3 vs. 10.9 vs. 7.8%) was higher in GWG below guidelines group than those in GWG above and within guidelines group, the risk of low Apgar score (6.4 vs. 3.0 vs. 2.8%) was higher in GWG above guidelines group than that in GWG below and within guidelines group (*P* < 0.05).

**Conclusion:**

Our findings demonstrated that GWG above and below guidelines, compared with GWG within guidelines, had a higher risk of adverse infant outcomes. Our findings also suggested that GWG below guidelines did not increase the risk for SGA, though SGA infants had more adverse outcomes among neonates born to mothers with GDM.

## Introduction

Gestational diabetes mellitus (GDM), a common pregnancy complication, affects 1–28% of all pregnancies. Maternal GDM contributes to short- and long-term health risks both for mothers (including polycystic ovarian syndrome, obesity, and type 2 diabetes) and children (including respiratory distress syndrome, hypoglycemia, hyperbilirubinemia, obesity, and metabolic syndrome) (1). The majority of newborns born to women with GDM are large-for-gestational-age (LGA) infants.

At present, the treatment of GDM includes dietary interventions and drugs. The current first-line therapy for GDM is dietary interventions, including lifestyle changes, weight management, physical activity, and medical nutrition therapy; 70–80% of GDM women were given dietary interventions to control blood glucose (2–4). Dietary interventions can reduce gestational weight gain (GWG). The targets of GWG during dietary interventions in many countries refer to the recommendations of the US Institute of Medicine (IOM), which were updated in 2009 based on pre-pregnancy body mass index (BMI) (5). GWG is an important antenatal factor, a few studies demonstrated that insufficient GWG (GWG below the IOM guidelines) during pregnancy was associated with a higher incidence of small-for-gestational-age (SGA) (6, 7). Studies demonstrated that SGA infants born to mothers with GDM had a higher risk of hypoglycemia and hyperbilirubinemia in infants and long-term cardiovascular hospitalizations in adulthood than LGA or appropriate-for-gestational-age (AGA) (8–10). However, it is not known if SGA associated with GDM is a risk factor for other perinatal complications. Moreover, the frequency of SGA born to mothers with GDM who were given dietary treatment has been reported to be as high as 11%, and this rate is increasing worldwide; the prevalence of insufficient GWG is also increasing (11, 12). Additionally, there is limited research on the association of insufficient GWG with SGA infants born to mothers with GDM. To better understand the relationship between insufficient GWG during the dietary intervention and perinatal outcomes, the aim of this study was to examine whether abnormal GWG will increase the frequency of SGA and the risk of adverse outcomes.

## Materials and methods

### Study design and participants

**Figure 1:**
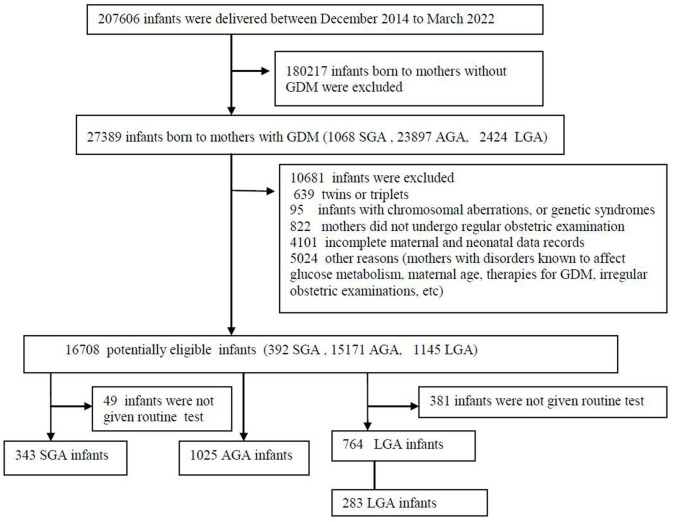
Flow diagram of study population. AGA, appropriate for gestational-age; GDM, gestational diabetes mellitus; LGA, large-for-gestational-age; SGA, small for gestational age.

This study was a population-based, retrospective case-control study and approved by the research ethical committee in Guangzhou Women and Children's Medical Centre of Guangzhou Medical University, Number 2014121402, date of approval 12 December 2014. The selection of participants is shown in Figure 1. All parents provided written informed consent, and the ethics committee approved this consent procedure. This study was conducted from December 2014 to March 2022.

The inclusion criteria were the followings: (1) mothers were diagnosed with GDM with singleton pregnancy and live birth; (2) mothers were older than 18 years; (3) mothers/infant pairs with complete maternal (delivery data, complication during pregnancy) and neonatal data (birth data, in-hospital outcomes); 4) all newborns were given routine examinations. The exclusion criteria: (1) infants presented with chromosomal aberrations, and genetic syndromes; (2) mothers with concomitant pathology that could affect glucose metabolism (such as diabetes, maturity-onset diabetes in young, polycystic ovarian syndrome, and uncontrolled thyroid during pregnancy); (3) mothers weren't given regular obstetric examinations. Only one birth infant for mother was included in our study. GDM was diagnosed according to guidelines for the diagnosis and treatment of GDM in China (13). The guidelines are similar to the American Diabetes Association and International Association of Diabetes and Pregnancy Study Group (IADPSG) guidelines (14). After the diagnosis of GDM, all GDM women who underwent regular obstetric examinations in hospitals received individualized dietary consultation with a dietitian. Individual recommendations for diet and exercise were based on the guidelines for the treatment of GDM in the China (13), and estimations of daily energy intake and nutrients were computed using a food database. Dietary recommendations were based on the following principles: restricting dietary intake of saturated fat and exchanging carbohydrate-rich foods with a medium-to-high glycaemic index for foods with a lower glycaemic index to reduce the glycaemic load. All women were recommended to participate in the aerobic and strength-conditioning exercises. The women were informed about weekly maternal GWG in late pregnancy, based on their pre-pregnancy BMI, independent of their GWG before GDM diagnosis as follows: gain of 12.5–18 kg for under-weight women (BMI < 18.5 kg/m^2^), 11.5–16 kg for normal women (BMI 18.5–24.9 kg/m^2^) and 7–11 kg for overweight women (BMI 25–29.9 kg/m^2^), 5–9 kg for obese women (BMI ≥30 kg/m^2^), and physical activity for at least 30 min/day were recommended.

All infants were divided into the following three groups: SGA infants born to mothers with GDM, AGA infants born to mothers with GDM, and LGA infants born to mothers with GDM. The diagnostic criteria of SGA infants: infants whose birth weight is below the 10th percentile at gestational age. The diagnostic criteria of AGA infants: infants whose birth weight is between the 10th and 90th percentile at gestational age. LGA was defined by a birth weight >90th percentile at gestational age. Classification of all newborns was defined according to the Fenton growth chart. GWG was defined as the difference between the final weight before delivery (within the last week before giving birth) and the pre-pregnancy weight (within 3 months before pregnancy). Based on the guidelines for maternal GWG of IOM, GWG was divided into the following three groups for analysis: GWG below guidelines (insufficient GWG), GWG within guidelines (sufficient GWG), and GWG above guidelines (excessive GWG). We performed matching according to neonatal gestational age (difference was ≤ 3 days), sex, and maternal age (difference was ≤ 3 years).

### Data collection

Each mother/infant pair's demographic data, intervention condition, and medical information were collected individually using medical records. All mother/infant pairs underwent structured medical examinations and physical examinations. The following data on siblings of included infants also were recorded: maternal blood glucose after a 75-g OGTT, glycated-hemoglobin (Hb) level, gestational age at delivery, sex, birth weight and height of newborn, and mode of delivery. The weight and length/height of mother/infant pairs were measured by trained nurses using standard anthropometric methods, and pre-pregnancy weight was obtained according to maternal self-report. BMI was calculated by dividing weight in kilograms by the square of height in meters. Early thrombocytopenia was defined as a platelet count of < 150 × 10^∧^9/L in the first 72 h of life. Fetal growth restriction (FGR) was determined by ultrasound, early FGR was defined as the gestational age was < 32 weeks and the following criteria were present: estimated fetal weight (EFW) or abdominal circumference (AC) below the 3rd percentile for the gestational age or absent end-diastolic flow in umbilical artery (UA); late FGR was defined as the gestational age was>32 weeks and the following criteria were present: EFW or AC below the 3rd percentile for the gestational age. Hypoglycemia was defined as blood glucose < 35 mg/dl or plasma glucose < 40 mg/dl within the first 48 h of life. Low Apgar score was defined as a 1-min Apgar score ≤ 7. Necrotizing enterocolitis (NEC) was diagnosed according to Bell criteria. Neonatal respiratory distress syndrome (NRDS) was diagnosed by (1) evidence of respiratory failure, (2) administration of exogenous pulmonary surfactant (3) radiographic evidence. Symptomatic polycythemia was defined as venous hematocrit >65 % or hemoglobin >220 g/L. Prematurity was defined as gestational age at birth of < 37 weeks. The presence of at least one neonatal complication (early thrombocytopenia, hypoglycemia, low Apgar score, NRDS, NEC, or polycythemia) was assessed.

Data entry was performed by a trained clerk and a supervisor. Data were cross-checked by the co-author (VF) for any errors and discrepancies. Data entered incorrectly will be examined and corrected by the supervisor after confirmation with the participants or their obstetric records. Any revision of the original data will be tracked in detail.

### Statistical analysis

Sample size calculation was performed using PASS 15. With 90% power and the assumption of relative risk = 2.0, we calculated that 256 SGA infants born to mothers with GDM were needed. Data were analyzed by using SPSS version 22 (SPSS, Chicago, IL, USA). Means (standard deviations) or median (range) was used to describe continuous variables; *t-*tests, ANOVA or kruskal-wallis test were used to analyze the differences in continuous variables. A two-sided chi-squared or Fisher's exact test was used for categorical variables presented as numbers and percentages. Binary logistic regression was used to determine risk factors for SGA infants born to mothers with GDM after adjusting for potential confounding variables. The association between birth weight and maternal GWG, pre-partum period BMI, and pre-pregnancy BMI was identified using Pearson's correlation analysis. Differences were considered statistically significant at a two-sided *P* value of < 0.05.

## Results

Overall, the final sample consisted of 1,651 mother/infant pairs. Of these, 343 SGA infants born to mothers with GDM, 1,025 AGA infants born to mothers with GDM, and 283 LGA infants born to mothers with GDM. There were 92 (5.6%) premature births, with 8 (2.3%) in the SGA infants born to mothers with GDM.

Relevant characteristics of mothers are presented in Table 1. We observed that GWG and Pre-partum BMI were lower in the SGA mothers with GDM group (11.7 ± 4.5 kg, 25.2 ± 3.1 kg/m^2^) than that in AGA (12.3 ± 4.6 kg, 26.3 ± 3.4 kg/m^2^) or LGA (14.0 ± 5.1 kg, 28.7 ± 3.9 kg/m^2^) mothers with GDM group (both *P* < 0.001). Preeclampsia was higher in SGA mothers with GDM than that in AGA mothers or LGA mothers (8.5 vs. 2.3 vs. 2.5%; *P* < 0.001). The incidence of primiparity (61.5%) and GWG below guidelines (50.4%) were highest in the SGA mothers group, and LGA mothers had the highest proportion of cesarean section (41.4%) and GWG above guidelines (39.2%).

**Table 1:** Clinical characteristics of the pregnant women (*N* = 1,651).

	**SGA mothers with GDM (*n* = 343)**	**AGA mothers with GDM (*n* = 1,025)**	**LGA mothers with GDM (*n* = 283)**	**P-value**
Age, mean (SD), years	31.6 (4.7)	31.9 (4.3)	32.7 (4.7)^h^	0.013
Rural residence No, (%)	292 (85.1)	870 (84.9)	253 (89.4)	0.148
Primiparity (primiparous) No. (%)	211 (61.5)	558 (54.4)^e^	100 (35.3)^h^	< 0.001
Pre-pregnancy BMI, median (range), kg/m^2^	20.2 (11.8-30.3)	21.1 (13.0-40.6)^f^	23.9 (11.9-36.0)^h^	< 0.001
Pre-pregnancy BMI < 18.5, No. (%)	91 (26.5)	168 (16.4)^f^	21 (7.4)^h^	< 0.001
Pre-pregnancy BMI 18.5–24.9, No. (%)	210 (61.2)	719 (70.1)^f^	169 (59.7)^h^	< 0.001
Pre-pregnancy BMI 25-29.9, No. (%)	39 (11.4)	117 (11.4)	75 (26.5)^h^	< 0.001
Pre-pregnancy BMI ≥ 30, No. (%)	3 (1.1)	21 (2.0)	18 (6.4)^h^	< 0.001
Pre-partum weight, median (range), kg	62.3 (44.7-92.6)	66.8 (44.5-124.1)^f^	74.1 (40.7-118.3)^h^	< 0.001
Pre-partum BMI, median (range), kg/m^2^	24.9 (19.1-34.0)	26.0 (18.8-44.9)^f^	28.3 (16.4-43.4)^h^	< 0.001
GWG, mean (SD), kg	11.7 (4.5)	12.3 (4.6)^f^	14.0 (5.1)^h^	< 0.001
GWG below guidelines, ^a^No. (%)	173 (50.4)	441 (43.0)^f^	59 (20.8)^h^	< 0.001
GWG within guidelines, ^a^No. (%)	121 (35.3)	398 (38.8)	113 (39.9)	0.414
GWG above guidelines, ^a^No. (%)	49 (14.3)	186 (18.1)	111 (39.2)^h^	< 0.001
Cesarean section, No (%)	142 (41.4)	339 (33.1)^f^	131 (46.3)^g^	< 0.001
Anemia, No (%)	28 (8.2)	61 (6.0)	24 (8.5)	0.182
Drug or illicit use, No (%)	1 (0.3)	2 (0.2)	1 (0.4)	0.872
Tobacco use, No (%)	2 (0.6)	11 (1.1)	1 (0.3)	0.277
Hypothyroidism, No (%)	7 (2.0)	14 (1.4)	6 (2.1)	0.542
ICP, No (%)	6 (1.7)	14 (1.4)	4 (1.4)	0.875
Oligohydramnios, No (%)	8 (2.3)	13 (1.3)	4 (1.4)	0.362
Pregnancy-induced hypertension, No, (%)	19 (5.5)	65 (6.3)	20 (7.1)	0.733
Placenta previa, No (%)	7 (2.0)	15 (1.5)	1 (0.4)	0.191
Placental abruption, No (%)	5 (1.5)	16 (1.6)	0 (0)^g^	0.110
Placental malformation, ^b^No (%)	10 (2.9)	14 (1.4)	3 (1.1)	0.103
Preeclampsia, ^c^No (%)	29 (8.5)	24 (2.3)^f^	7 (2.5)^h^	< 0.001
Single umbilical artery, No (%)	2 (0.6)	2 (0.2)	0 (0)	0.297
Uterine malformation, ^d^No (%)	3 (0.9)	3 (0.3)	0 (0)	0.161

AGA, appropriate for gestational-age; BMI, body mass index; GDM, gestational diabetes mellitus; GWG, gestational weight gain; ICP, intrahepatic cholestasis of pregnancy; LGA, large-for-gestational-age; SD, standard deviations; SGA, small for gestational age.

^a^GWG below guidelines (insufficient GWG) was defined as weight gain during gestation of < 12.5 kg in underweight women, < 11.5 kg in normal weight women, < 7 kg in overweight women (BMI 25.0–30.0 kg/m^2^), and < 5 kg in obese women (≥30.0 kg/m^2^). GWG above guidelines (excessive GWG), which was defined as a weight gain during gestation of >18 kg in underweight women, >16 kg in normal weight women, >11.5 kg in overweight women, and >9 kg in obese women. Women with GWG within IOM recommendations were classified as having within GWG guidelines (sufficient GWG).

^b^placental malformation contains velamentous placenta and battledore placenta.

^c^pre-eclampsia is defined as new-onset hypertension [systolic blood pressure ≥140 mmHg or diastolic blood pressure ≥90 mmHg] and new-onset proteinuria [300 mg of protein in 24 h or a urine protein/creatinine ratio of 0.3 mg/dL] after 20 weeks gestation or, in the absence of proteinuria, new-onset hypertension with new-onset thrombocytopenia, renal insufficiency, impaired liver function, pulmonary edema, or cerebral or visual disturbances.

^d^uterine malformation contains uterus hypoplasia, monocular uterus, double uterus, biangular uterus, mediastinal uterus, and arch uterus.

^e^*P* < 0.05, SGA mothers with GDM vs. AGA mothers with GDM.

^f^*P* < 0.01, SGA mothers with GDM vs. AGA mothers with GDM.

^g^*P* < 0.05, SGA mothers with GDM vs. LGA mothers with GDM.

^h^*P* < 0.01, SGA mothers with GDM vs. LGA mothers with GDM.

Table 2 shows the clinical characteristics of the infants. The frequency of SGA infants who were siblings (41.7 vs. 4.3 vs. 1.9%) and composite of complications (19.2 vs. 12.0 vs. 11.7%) were higher in SGA infants than in those in AGA or LGA infants group (both *P* < 0.01). The risk of symptomatic polycythemia was more frequent in LGA infants than that in the AGA or SGA infants group (3.2 vs. 0.3 vs. 0.6%, *P* < 0.001). The risk of early thrombocytopenia was increased in SGA infants compared to that in AGA infants (1.7 vs. 0.2%). However, there were no significant differences in the risk of Apgar score, hypoglycemia, hypoglycemia encephalopathy, NEC, NRDS, or perinatal death among the three groups (all *P* > 0.05).

**Table 2:** Clinical characteristics of the infants (*N* = 1,651).

	**SGA (343)**	**AGA (1025)**	**LGA (283)**	**P-value**
Gestational age, median (range), weeks	38.3 (33.3–40.9)	38.7 (28.9–41.1)^a^	39.0 (28.1–41.1)^c^	< 0.001
Sex (boys/girls)	182:161	532:493	165:118	0.161
Birth weight (BW), mean (SD), g	2393.2 (236.6)	3097.6 (325.9)^a^	4050.1 (360.9)^c^	< 0.001
Siblings who were SGA, No, (%)	60 (41.7)	23 (4.3)^a^	4 (1.9)^c^	< 0.001
**Apgar score**				
1 min low ( ≤ 7), No (%)	10 (2.9)	37 (3.6)	7 (2.5)	0.583
1 min low ( ≤ 3), No (%)	3 (0.9)	2 (0.2)	1 (0.4)	0.194
Early thrombocytopenia, No (%)	6 (1.7)	2 (0.2)^a^	3 (1.1)	0.006
Birth glucose, mean (SD), mmol/l	3.6 (0.8)	3.8 (0.9)^a^	3.5 (0.6)	0.001
Hypoglycemia, No (%)	37 (10.8)	80 (7.8)	18 (6.4)	0.103
Hypoglycemia encephalopathy, No (%)	1 (0.3)	1 (0.1)	0 (0)	0.545
NEC, No (%)	3 (0.9)	3 (0.3)	0 (0)	0.161
NRDS, No (%)	2 (0.6)	6 (0.6)	0 (0)	0.435
Perinatal death, No (%)	1 (0.3)	2 (0.2)	0 (0)	0.686
Polycythemia, No (%)	2 (0.6)	3 (0.3)	9 (3.2)^b^	< 0.001
Composite of complications, No (%)	66 (19.2)	123 (12.0)^a^	33 (11.7)^c^	0.002

AGA, appropriate for gestational-age; BMI, body mass index; GDM, gestational diabetes mellitus; LGA, large-for-gestational-age; NEC, necrotizing enterocolitis; NRDS, neonatal respiratory distress syndrome; SD, standard deviations; SGA, small for gestational age.

^a^*P* < 0.01, SGA infants vs. AGA infants.

^b^*P* < 0.05, SGA infants vs. LGA infants.

^c^*P* < 0.01, SGA infants vs. LGA infants.

Pearson's correlation analysis was used to determine the correlation between maternal factors and birth weight, and the results are shown in Table 3. The results illustrated that GWG, prepregnancy BMI, and prepartum period BMI were significantly weakly correlated with infants' birth weight (Pearson correlation coefficient 0.129, 0.113, and 0.170; all *P* < 0.01).

**Table 3:** Relationships between birth weight and gestational weight gain, prepartum period BMI, pre-pregnancy BMI in infants born with GDM mother (*N* = 1,651).

	**Pearson correlation coefficient(r)**	**P-value**
Prepartum period BMI	0.170	< 0.01
Prepregnancy BMI	0.113	< 0.01
Gestational weight gain	0.129	< 0.01

BMI, body mass index; GDM, gestational diabetes mellitus.

Neonates were further grouped by the guidelines for maternal GWG of IOM as follows: GWG below guidelines, GWG within guidelines, and GWG above guidelines. Table 4 shows the influence of GWG on perinatal outcomes. 673 (40.8%) women had GWG below guidelines, 346 (21.0%) had GWG above guidelines, and 632 (38.2%) had GWG within guidelines. We observed that the risk of SGA (25.7 vs. 19.1 vs. 14.2%) and FGR (15.3 vs. 10.9 vs. 7.8%) was higher in GWG below guidelines group than those in GWG above and within guidelines group. However, the risk of low Apgar score (6.4 vs. 3.0 vs. 2.8%) was higher in GWG above guidelines group than that in GWG below or within guidelines group (*P* < 0.05). There were no significant differences in adverse women outcomes, early thrombocytopenia, NEC, hypoglycemia, or NRDS among these three groups.

**Table 4:** Maternal and neonatal outcomes by maternal weight gain category (*N* = 1,651).

	**GWG below guidelines^a^ (*n* = 673)**	**GWG within guidelines^a^ (*n* = 632)**	**GWG above guidelines^a^ (*n* = 346)**	**P-value**
Cesarean delivery, No (%)	223 (33.1)^b^	249 (39.4)	124 (35.8)	0.062
FGR, No (%)	103 (15.3)^b^	69 (10.9)	27 (7.8)	0.001
Pregnancy-induced, hypertension No (%)	51 (7.6)	35 (5.5)	18 (5.2)	0.203
Preeclampsia, No (%)	28 (4.2)	25 (4.0)	7 (2.0)	0.194
Early thrombocytopenia, No (%)	7 (1.0)	3 (0.5)	1 (0.3)	0.284
Hypoglycemia, No (%)	58 (8.6)	54 (8.5)	23 (6.6)	0.505
Low APGAR, No (%)	20 (3.0)	18 (2.8)	22 (6.4)^e^	0.010
NEC, No (%)	3 (0.4)	2 (0.3)	1 (0.3)	0.897
NRDS, No (%)	5 (0.7)	2 (0.3)	1 (0.3)	0.455
Symptomatic polycythemia, No (%)	2 (0.3)	5 (0.8)	7 (2.0)	0.017
SGA, No (%)	173 (25.7)^c^	121 (19.1)	49 (14.2)^d^	< 0.001

FGR, fetal growth restriction; NEC, necrotizing enterocolitis; NRDS, neonatal respiratory distress syndrome; GWG, gestational weight gain; SGA, small for gestational age.

^a^GWG below guidelines (insufficient GWG) was defined as weight gain during gestation of < 12.5 kg in underweight women, < 11.5 kg in normal weight women, < 7 kg in overweight women (BMI 25.0–30.0 kg/m^2^), and < 5 kg in obese women (≥30.0 kg/m^2^). GWG above guidelines (excessive GWG), which was defined as a weight gain during gestation of >18 kg in underweight women, >16 kg in normal weight women, >11.5 kg in overweight women, and >9 kg in obese women. Women with GWG within IOM recommendations were classified as having within GWG guidelines (sufficient GWG).

^b^*P* < 0.05, GWG below guidelines vs. GWG within guidelines.

^c^*P* < 0.01, GWG below guidelines vs. GWG within guidelines.

^d^*P* < 0.05, GWG above guidelines vs. GWG within guidelines.

^e^*P* < 0.01, GWG above guidelines vs. GWG within guidelines.

Next, multivariate binary logistic regression analysis was performed to identify factors associated with SGA, and the results are shown in Table 5. Adjusting for other confounding variables, the following factors were associated with SGA: preeclampsia (AOR 3.12, 95% CI [1.34–7.30]) and siblings who were SGA (AOR 18.06, 95% CI [10.83–30.13]).

**Table 5:** Binary logistic analysis for the associations between maternal complications and SGA infants (*N* = 1,651).

**Risk factors**	**Adjusted odds ratio^a^**	**95% confidence interval**	**P-value**
GWG below guidelines	1.49	0.92–2.21	0.12
Preeclampsia	3.12	1.34–7.30	< 0.01
Siblings who were SGA	18.06	10.83–30.13	< 0.01

^a^Adjusted odds ratio (AOR) controlled for covariates including sex, maternal age, rural residence, pre-pregnancy BMI, and primiparity.

GWG, gestational weight gain; SGA, small for gestational age.

To further evaluate the effect of risk factors for SGA on GWG among women with GDM, mothers were further grouped by risk factors for SGA as follows: GDM mothers with and without preeclampsia, GDM mothers with and without siblings who were SGA. However, groups did not differ significantly in pre-pregnancy BMI, GWG, the incidence of GWG below guidelines, GWG within guidelines, and GWG below guidelines (all *P* > 0.05) (Table 6).

**Table 6:** The comparison of gestational weight gain and pre-pregnancy BMI between GDM mothers with and without risk factors (*N* = 1,651).

		**GDM mothers with risk factor^a^**	**GDM mothers without risk factor**	**P-value**
GDM mothers with and without preeclampsia		*N* = 60	*N* = 1591	
	Age, mean (SD), years	33.2 (5.3)	32.0 (4.5)	0.052
	SGA, No, (%)	29 (48.3)	314 (19.7)	< 0.001
	Pre-pregnancy BMI, median (range), kg/m^2^	22.8 (3.2)	21.8 (3.8)	0.149
	GWG, mean (SD), kg	11.7 (4.8)	12.9 (8.0)	0.377
	GWG below guidelines, No. (%)	21 (35.0)	652 (40.9)	0.355
	GWG within guidelines, No. (%)	28 (46.7)	604 (38.0)	0.173
	GWG above guidelines, No. (%)	11 (18.3)	335 (21.1)	0.611
GDM mothers with and without siblings who were SGA		*N* = 87	*N* = 1564	
	Age, mean (SD), years	32.6 (4.8)	31.9 (4.5)	0.157
	SGA, No (%)	60 (69.0)	283 (18.1)	< 0.001
	Pre-pregnancy BMI, median (range), kg/m^2^	21.8 (3.2)	21.7 (3.8)	0.608
	GWG, mean (SD), kg	12.3 (4.8)	12.5 (4.8)	0.750
	GWG below guidelines, No. (%)	29 (33.3)	644 (41.2)	0.147
	GWG within guidelines, No. (%)	37 (42.5)	595 (38.0)	0.402
	GWG above guidelines, No. (%)	21 (24.2)	325 (20.8)	0.454

^a^Risk factor is preeclampsia or siblings who were SGA.

BMI, body mass index; GDM, gestational diabetes mellitus; GWG, gestational weight gain; SD, standard deviations; SGA, small for gestational age.

## Discussion

We conducted a population-based, case-control study to explore the association between dietary intervention and the frequency of SGA. The main results of our study suggested the following: (1) siblings who were SGA and pre-eclampsia could be the risk factors for SGA infants born to mothers with GDM, infants' birth weight is also correlated with GWG, prepregnancy BMI, and prepartum period BMI; (2) compared to AGA or LGA, SGA infants born to mothers with GDM have more adverse perinatal outcomes; (3) GWG above and below guidelines increased the risk of adverse infant outcomes.

Dietary advice and exercise interventions are the first-line therapy for pregnant women with GDM. Dietary advice and exercise interventions alone for the prevention of GDM have been widely assessed; dietary and exercise interventions have been proven to reduce GWG in these studies (15, 16). So international guidelines recommend that pregnant women with GDM participated in aerobic and strength-conditioning exercises for 30 min at least 5 days per week along with medical nutritional therapy (17, 18). The main purpose of dietary interventions for pregnant women with GDM is to prevent macrosomia due to its associated risks (19, 20). However, some findings suggested that preventing SGA and related complications should be just as important in the care of pregnant women with GDM (21–23). The birth rate of SGA infants was similar to that of LGA infants in these studies; nevertheless, perinatal complications (20.1%) and mortality (1.6%) were worse in the SGA group than in the AGA or LGA groups (11, 24, 25). Our findings were in agreement with these previous reports. In normal pregnancies, the risk of perinatal asphyxia, hypoglycemia, polycythemia, thrombocytopenia, and other neonatal complications is also higher in SGA neonates than in AGA or LGA neonates (26–28).

Dietary advice and exercise interventions can reduce GWG, Pearson's correlation analysis also showed that GWG was correlated with infants' birth weight in our study; but whether they can increase the incidence of SGA remains controversial (6, 15). IOM recommended different targets for an adequate GWG depending on the pre-pregnancy BMI in 2009. In China, insufficient GWG (GWG below guidelines) occurs in 33.9% of pregnant women with GDM (29). In our study, 40.8% of GDM women presented with a total GWG below the IOM guidelines; this variation may be due to rigorous lifestyle improvements. Recent studies have shown that insufficient GWG results in increased odds of preterm birth and SGA in normal pregnancies and women with GDM (30–33). However, Gou et al. (34) showed that insufficient GWG was not the risk factor for SGA. Adjusting for other confounding variables, the results of logistic regression analysis also showed that there was no positive correlation between SGA and insufficient GWG in our study. The inconsistency may be due to the different study populations, the calculation method of GWG and the adjusted confounding variables. Therefore, more and larger randomized controlled trials are needed to assess the relationship between insufficient GWG and SGA in Chinese.

Moreover, some previous studies reported that excessive GWG (GWG above guidelines) had increased the odds of hypertensive disorders of pregnancy, cesarean delivery, macrosomia, and LGA (35, 36); but the effects of excessive GWG on adverse infant outcomes have not been analyzed in detail in these studies. GDM women with excessive GWG increased the risk for low Apgar score (6.4%) in our study compared to that in GWG below or within guidelines group.

The recurrence risk among siblings is widely used to measure shared genetic contributions. The frequency of SGA siblings in SGA infants born to mothers with GDM group was 10-fold higher than that in the AGA group in our study. Therefore, our findings demonstrated that genetic factors and preeclampsia were risk factors for SGA infants born to mothers with GDM. Recently, Spanish scholars indicated that smoking and neonate prematurity were also risk factors for these infants (11); however, we did not find this result in our study.

Our study presents novel information, not previously reported, as we considered the frequency of siblings who were SGA, neonatal early thrombocytopenia, and abnormal GWG among GDM women. Our findings are also helpful for clinicians to design more accurate care programs for pregnant women with GDM. Our study also has several limitations. First, the number of SGA infants born to mothers with GDM may be small. Second, the other limitation is reliance on self-reported pre-pregnancy weight. Third, the time for the blood routine examination and blood glucose test was varied for each infant, which might affect the results. Fourth, it was a retrospective observational study; therefore, selection and information bias cannot be excluded.

## Conclusion

In conclusion, our findings suggested that GWG below guidelines did not increase the risk for SGA, though SGA infants had more adverse outcomes among neonates born to mothers with GDM. However, SGA infants born to mothers with GDM remain a major concern. Moreover, we identified that genetic factors and pre-eclampsia could be the risk factors for SGA infants born to mothers with GDM. Our research also demonstrated that GWG above and below guidelines, compared with GWG within guidelines, had a higher risk of adverse infant outcomes. These findings suggest that it is necessary to maintain a reasonable GWG among pregnant women with GDM to reduce adverse perinatal complications.

## Data availability statement

The original contributions presented in the study are included in the article/supplementary material, further inquiries can be directed to the corresponding authors.

## Ethics statement

The studies involving human participants were reviewed and approved by Ethical Committee in Guangzhou Women and Children's Medical Centre of Guangzhou Medical University, Number 2014121402, date of approval 12 December 2014. The patients/participants provided their written informed consent to participate in this study. Written informed consent was obtained from the individual(s), and minor(s)' legal guardian/next of kin, for the publication of any potentially identifiable images or data included in this article.

## Author contributions

DH, ML, BX, WZ, and JC designed the study, designed the data collection instruments, collected data, carried out the initial analyses, drafted the initial manuscript, and created the tables and figures. SC, YX, and JC conceptualized and designed the study, coordinated and supervised data collection, and helped draft the initial manuscript. HL, LW, PP, DY, YY, and JY designed the data collection instruments, collected data, and conducted the initial analyses. All authors contributed to the manuscript's critical revision, read, and approved the submitted version.
