# Corrigendum: The association of insufficient gestational weight gain in women with gestational diabetes mellitus with adverse infant outcomes: a case-control study

**DOI:** 10.3389/fpubh.2023.1349218

**Published:** 2023-12-22

**Authors:** Dabing Huang, Mulin Liang, Bin Xu, Shan Chen, Yan Xiao, Hui Liu, Dan Yin, Jun Yang, Ling Wang, PianPian Pan, Yihui Yang, Wei Zhou, Juncao Chen

**Affiliations:** ^1^Department of Neonatology, Guangzhou Women and Children's Medical Centre, Guangzhou Medical University, Guangzhou, China; ^2^Department of Neonatology, The Fifth Affiliated Hospital of Southern Medical University, Guangzhou, China; ^3^Medical Department, The First People's Hospital of Chenzhou, Chenzhou, China; ^4^Rehabilitation Medicine Center, The First Dongguan Affiliated Hospital, Guangdong Medical University, Dongguan, China; ^5^Advanced Institute of Natural Sciences, Beijing Normal University at Zhuhai, Zhuhai, China

**Keywords:** dietary intervention, gestational diabetes mellitus, gestational weight gain, neonatal complications, risk factors, small-for-gestational age

In the published article, there was an error in the **Funding** statement. The grant number for the Research foundation of Guangzhou Women and Children's Medical Center for Clinical Doctor and Basic research program and applied basic research project of Guangzhou Science and Technology Bureau was erroneously displayed as “(2210124-03)”. This grant number should have been written as “(202102010329)”. The corrected **Funding** statement appears below:

“This article was supported by Research foundation of Guangzhou Women and Children's Medical Center for Clinical Doctor and Basic research program and applied basic research project of Guangzhou Science and Technology Bureau (202102010329).”

The authors apologize for this error and state that this does not change the scientific conclusions of the article in any way. The original article has been updated.

